# The Treatment of General Peritonitis

**Published:** 1912-03-16

**Authors:** Leonard A. Bidwell

**Affiliations:** Surgeon to the West London Hospital.


### THE TREATMENT OF GENERAL PERITONITIS.

By LEONARD A. BIDWELL, F.R.C.S., Surgeon to the West London Hospital.

(A Lecture delivered at the Post-Graduate College.)

Although this lecture chiefly refers to treatment, I will first call your attention to the common causes of acute general peritonitis. As you are all aware, disease of the vermiform appendix is by far the most common cause, and is accountable for over 60 per cent. of cases of acute general peritonitis which come under the care of a surgeon. Of the remaining cases, about 20 per cent. are due to perforation of the alimentary tract, 10 per cent. owe their origin to diseases of the pelvic viscera, and 10 per cent. to miscellaneous causes.

### Some Less Common Causes.

I shall therefore devote the greater part of the time at my disposal to the consideration of general peritonitis due to appendicitis, but before doing this I would mention some of the other causes of general peritonitis with which I have recently met. First, with regard to perforations of the alimentary tract, the most common are those of a gastric or duodenal ulcer, the former being more prevalent in females and the latter in males. The other perforations I have met with include: (1) Perforations of the colon in cases of ulcerative colitis; here, however, the peritonitis has remained local in nearly every case; (2) perforation of a stercoral ulcer of the sigmoid after cicatricial contraction of the bowel following removal of the rectum for carcinoma; this occurred in two cases, in one of which the inflammation was general and proved fatal; in the other the peritonitis was local, and the patient recovered after a laparotomy and closure of the perforation; (3) perforation of typhoid ulcers, in which the prognosis is very bad; (4) perforation of a tuberculous ulcer; and, lastly (5), penetration of the rectum by a bullet wound, which produced fatal general peritonitis. A considerable number of cases of general peritonitis were due to perforation of a pyosalpinx, usually of gonorrhœal origin. With regard to miscellaneous causes, I have had two or three cases of general peritonitis from perforation of a gangrenous gall-bladder, and one from rupture of a subperitoneal abscess. With regard to the symptoms of acute general peritonitis, pain is nearly always present, and is usually very acute at first, but when a large amount of pus has formed the pain often becomes less severe, or even disappears altogether, and in cases of perforation of a typhoid ulcer it may be absent, as the patient is often delirious.

Vomiting is a practically constant symptom, and after the first, the vomit is usually pumped up in large quantities without any effort; its character is bilious at first, but afterwards becomes brown and acrid smelling, and is often described as feculant. This, however, is probably an inaccurate description, as the odour and colour are often due to the presence of altered blood, which is poured out into the intestine from the inflamed mucous membrane in cases of intense peritonitis. A rigor is not uncommon, but is not constant. The temperature is nearly always raised at the commencement of peritonitis, though it often falls to normal, or even lower afterwards. The pulse is nearly always increased in frequency, and is thin in volume. The aspect of the patient is characteristic of some severe lesion; he is anxious, his face is drawn and often dusky in colour, and the eyes are sunken; later, the lips become dry and coated, and the so-called "Facies Hippocratica" is produced. With regard to the abdomen, the first sign is that it does not move on respiration; sometimes this only applies to the lower part. It is not distended at first, but is rigid; later it becomes distended and tympanitic. There is often shifting dulness, due not so much to the presence of free peritoneal fluid as to the movements of intestinal contents. Tenderness is often marked. The rectal mucous membrane is congested, and examination gives great pain. Micturition is often painful. In peritonitis due to perforation, free gas is present in the peritoneal cavity, and gives rise to the symptom of obliteration of the liver dulness. This sign, however, is only of value when discovered in the early stage of peritonitis, while the abdomen is still contracted, since a distended transverse colon produces diminution of liver dulness. The symptom was present in about half the cases. Free gas is not usually found in general peritonitis due to perforation of an appendix. I have, however, detected it in large collections of pus, especially when situated in the pelvis.

### The Diseased Appendix.

To turn now to the commonest cause of general peritonitis, namely, a diseased appendix, it is interesting to consider the condition of the organ in which it is likely to occur. Roughly speaking, there are three main types of appendicitis—(1) a form in which the appendix becomes inflamed, but which subsides under treatment, simply leaving adhesions; this includes cases in which, after a stricture, pus is formed in the distal portion; (2) a form in which some minute leak occurs, but gets shut off by adhesions from the general peritoneal cavity, so a local abscess forms; (3) a form in which very frail adhesions form round a slight leak of the appendix and peritonitis spreads locally; and (4) the form in which the appendix becomes gangrenous, or ruptures without preliminary formation of adhesions, and acute general peritonitis immediately supervenes. It is, however, not only in the latter type of case that we meet with general peritonitis. A localised abscess may burst and produce general peritonitis, or the localised spreading peritonitis may become general. In my experience, the worst cases of general peritonitis are those in which a large abscess bursts into the general peritoneal cavity. In these cases such a terrific dose of toxins is absorbed at once by the healthy peritoneum that there is little hope of saving the patient by operation.

The cause of the rapidly fatal termination of acute general peritonitis is toxæmia—(1) from absorption from the peritoneum, and (2) from absorption from the interior of the intestines. (1) With regard to absorption from the peritoneum, it is a curious fact that the most active and rapid absorption takes place from the peritoneum in the upper part of the abdomen, and the least active absorption from the pelvis. A large collection of pus may accumulate and remain in the pelvis for some days without producing any serious symptoms, whereas a collection in the upper part or in the subphrenic region rarely occurs without very grave and urgent symptoms. When pus has accumulated in any part of the peritoneal cavity, the peritoneum very soon loses its power of absorption of toxins; this accounts for the mildness of the constitutional effects met with in cases of localised abscess.

(2) *The absorption from the interior of the intestines.*—Although bacilli coli communis exist in great quantities in the lower ileum and in the large intestine during health, the changes which take place in the nutrition of the intestine during peritonitis afford a different medium for these bacilli, and this changes their comparatively harmless nature, causing them to secrete a highly toxic ptomaine. The damaged condition of the walls of the gut caused through the inflammation favours the easy and rapid absorption of these toxins. The general system is attacked by a double invasion of toxins, and unless this is stopped death must occur. The possibility of checking absorption from the intestines by producing diarrhœa is the basis of the method of giving saline aperients in general peritonitis.

### Surgical Treatment.

Modern surgery, however, teaches that the most hopeful way of treating general peritonitis is by operation, and the aim of the surgeon is to remove first the cause of the peritonitis and then all source of toxin infection. This can only be done by opening the abdomen and wiping away or flushing away all pus within the peritoneal cavity, and by emptying the portions of the bowel which are distended with the virulent culture of bacilli coli communis. Our first indication in general peritonitis is not to give morphia, since it produces intestinal paralysis, and prevents nature's attempt to get rid of the toxins by diarrhœa. Our second indication is to open the abdomen as soon as possible. In the case of general peritonitis from rupture of an appendix the prognosis will almost entirely depend upon the length of time which has elapsed since perforation has taken place. If you are so fortunate as to see the case within six hours of the onset of peritonitis you will probably save the patient; after this time the prognosis becomes more grave with every hour's delay.

We generally have some indication of the cause of the peritonitis by the situation of the pain, the presence of some local rigidity, or by the history of previous attacks of appendicitis. At any rate, we shall probably find that the most intense symptoms are at the lower right side of the abdomen. One word as to the choice of an anæsthetic. In children, at any rate, chloroform is contra-indicated, as it induces acetonuria, and this often has an injurious effect. Ether by the open method is to be preferred, or, failing this, a mixture of ether with chloroform. In adults, the question of spinal analgesia should be considered. It has the great advantage of not producing or increasing vomiting. In cases where the patient is greatly collapsed an injection of one pint of saline containing ℥i of brandy should be given per rectum before the operation, or when diarrhœa accompanies the collapse it is desirable to start intravenous infusions into the median cephalic vein at the commencement of and to continue it during the operation, infusing as much as five pints. Let me here remind you that the strength of the saline solution for intravenous injection should be $1\frac{1}{2}$ dr. to the pint, and that the solution should be sterilised by boiling.

### The Different Incisions.

The abdomen should be opened by an incision of sufficient length thoroughly to explore the parts without any risk of bruising the inflamed intestines. When it is expected that peritonitis is due to perforation of the appendix, an incision should be made at the outer edge of the right rectus muscle. When the Fallopian tubes are suspected of causing the trouble, an incision should be made at the inner edge of the rectus muscle. When the mischief is probably due to perforation of a gastric or duodenal ulcer, the incision should be made at the inner edge of the right rectus muscle above the umbilicus. When it is quite uncertain which organ is the cause of the peritonitis, it is best to make a small incision at the outer edge of the rectus muscle opposite to the umbilicus, and then to enlarge this either upwards or downwards when the cause has been detected. After opening the abdomen, the cause of the peritonitis must be searched for and removed. A perforated or gangrenous appendix must always be removed at once. A perforated gastric, duodenal, or other ulcer must be closed, and a suppurating tube removed. The question of drainage will then have to be decided. When only a small quantity of pus is present, especially when peritonitis is due to a perforation in the upper part of the intestinal tract, or of a pyosalpinx, wiping away of the pus will be sufficient, without drainage. In cases where there is considerable amount of purulent effusion, especially when this is caused by perforation of the appendix or of the lower part of the intestine, irrigation will be necessary to cleanse the peritoneum. Before doing irrigation, it is, however, necessary to provide efficient drainage, since it is extremely likely that irrigation, carried out before drainage had been provided, would drive the poison further into the peritoneal cavity.

### Draining the Pelvis.

The drainage required consists of openings in each loin and into the pelvis. The lateral drains are necessary to prevent any accumulation of fluid in the subphrenic spaces, and are inserted in the following way: An incision is made below the tip of the last rib, through the skin and fascia. A large pair of forceps is then pushed through the muscles on to a finger introduced into the peritoneal cavity. The forceps is then made to penetrate the peritoneal cavity, and is brought out of the anterior incision. A drainage tube is then seized and dragged out of the lateral incision on withdrawing the forceps. Drainage of the pelvis is best effected by a rectal or vaginal drain. The rectal drain is inserted by pushing a pair of large clamp forceps into the pelvis and feeling for its point by a finger introduced into the rectum. When the point of the forceps is felt, it is pushed through the wall of the rectum and made to project outside the anus. A large tube is then seized by the forceps, dragged through the anus and through the wound in the wall of the rectum into the pelvis. The tubes should be of large size. It is better to have a large tube in for a short time than a small one for a longer time. When lateral and pelvic drainage has been provided, the abdomen can be thoroughly flushed out with saline solution.

### Avoid Antiseptics.

No antiseptic should ever be used for flushing out the peritoneum. Not only is there a risk of a poisonous dose of the antiseptic being absorbed by the peritoneum, but there is a certainty of the peritoneal cells being damaged by the chemical. When the peritoneum has been thoroughly flushed out, the intestine must be examined with a view to deciding whether it would be advisable to open it and empty it of its contents. This course should be reserved for cases in which peritonitis has existed for some considerable time, and in which there is paralysis of the intestine. A coil of small intestine is brought outside the abdomen, and after being packed round with gauze swabs, an incision about half-inch long is made in its convexed order, the contents of the loop are expressed, and other coils are "milked" into this one by placing the hand inside the abdomen. Some surgeons introduce a stiff tube into the intestine, and gradually work many feet of the gut on to this tube. This, however, is liable to cause more damage to the mucous membrane. When the intestine has been emptied in this way, it is advisable to wash it out with saline solution and then to close the opening with a double row of Lembert's stitches. The anterior abdominal incision should then be closed, leaving an interval in its centre for drainage. For this purpose it is better to use catgut stitches rather than those of silk or thread, as there is a risk of the latter becoming infected and keeping up a discharge from the wound.

### Position in After-Treatment.

In the after-treatment of these cases, the position of the patient is most important. It is always best to put the patient in the half-sitting or Fowler's position as soon as he has recovered from the anæsthetic. The use of this position prevents the accumulation of any discharge in the subphrenic spaces, and insures that it will track down into the pelvis, where it will escape by the rectal drain. Some form of stimulant is sure to be necessary, and as soon as post anæsthetic sickness has stopped brandy and champagne can be given by the mouth. The stimulant, however, which is of the greatest value is pituitary extract. This should be injected into a muscle in 1 c.c. doses every four hours for the first two days. Strychnine is not of any particular value, and may increase shock. The nourishment of the body must be kept up by the introduction of saline solution. This can be given by the rectum in half-pint enemata every four hours. It is introduced by the side of the rectal drainage-tube, and the presence of this tube does not, as a rule, interfere with the giving of salines. It is advisable to add some nourishment to the salines, the simplest method being the addition of half an ounce of glucose to each enema. The salines are continued until three pints of fluid are taken by the mouth within twenty-four hours. When some proportion of this quantity is taken by the mouth, the amount given by the rectum is diminished accordingly. When salines cannot be retained by the rectum the injections should be made subcutaneously. When rectal drainage has not been employed, continuous rectal irrigation or proctolysis may be used. Not only is this of value in keeping up the nutrition of the body, but it facilitates the excretion of toxins from the intestines. It is adopted as a routine treatment by many surgeons. The next most important point in after-treatment is the management of the bowels, and the success of a case will often depend upon obtaining a satisfactory action of them. On the day after the operation, calomel may be given in half-grain doses every hour until three or four grains have been taken. In addition to this, sulphate of magnesia combined with strychnine and belladonna may be given every four hours, provided there is no sickness. When, however, the patient is sick it is better to get the bowels open by enemata. Amongst these we may employ one containing magnesium sulphate, ox gall, or turpentine. It will be found that the exhibition of pituitary favours the re-establishment of peristaltic action of the bowels. As there is toxic absorption from the intestines, due to the presence there of coli bacilli, it is sometimes advisable to give doses of lactic acid bacilli.

### The Question of Diet.

With regard to food, no milk should be given for the first three days, as this not only causes the formation of clots in the stomach but favours constipation. The most important variety of food which is required is sugar; not only is this nourishing, but it favours the growth of lactic acid bacilli. It is given in the form of raisin tea. This is made by pouring boiling water upon raisins, and consists of a solution of glucose with a muscatel flavour. In addition to this, barley water and albumen water and some of the patent foods, such as Benger's or Hygiama, may be given. Beef-tea and meat juice are not of any great value except as stimulants. Stimulants can be given freely. Morphia may be given for the first day, but should always be combined with atropin. The drainage tubes should be removed after forty-eight hours, and after their removal a cigarette drain should be placed in each incision. The rectal drain is also removed on the third day. In a case which is going to recover the general condition becomes satisfactory after the bowels have acted freely.

